# Editorial: Human movement and motor control in the natural environment

**DOI:** 10.3389/fbioe.2023.1210173

**Published:** 2023-05-19

**Authors:** Maurice Mohr, Peter Federolf, Gert-Jan Pepping, Thorsten Stein, Steven van Andel, Gillian Weir

**Affiliations:** ^1^ Department of Sport Science, University of Innsbruck, Innsbruck, Austria; ^2^ School of Behavioural and Health Sciences, Australian Catholic University, Brisbane, QLD, Australia; ^3^ Institute of Sports and Sports Science, Karlsruhe Institute of Technology (KIT), Karlsruhe, Baden-Württemberg, Germany; ^4^ IJsselheem Foundation, Kampen, Netherlands; ^5^ New York Yankees, Tampa, FL, United States

**Keywords:** ecological valididy, motor learning and control, wearable sensor, movement analysis, principal compoment analysis, inertial motion capture, coaching (athletics), motor competence assessment

## Introduction

Studies in human movement science are typically motivated by the underlying goal to improve human performance and/or quality of life, e.g., through more efficient or effective training and rehabilitation of the movement system over the lifespan. While the respective experimental studies usually take place in laboratory environments, their conclusions and predictions are meant to inform training and rehabilitation in the real world, e.g., on the playing field, on the sidewalk, or at the work place. In the natural sciences, this is the traditional approach: we simplify problems so that we can design rigorous laboratory studies that follow the scientific method to then draw strong conclusions about the real world. In human movement science, however, simplifying the problem may more often than not lead to conclusions that are limited, if not invalid, outside of the laboratory, i.e., they lack ecological validity. This is because the human movement system has evolved in constant interaction with the highly complex, natural environment, which is often neglected in the simplified laboratory environment. As a result, any systematic assessment of the movement system must be a balance between creating conditions that allow to study an effect of interest while ensuring that the observed behavior is still relevant in the real world. With the advent of more advanced technologies to measure unconstrained human movement, tipping this balance towards the real world has become more feasible, however, the complexity of the obtained data often requires equally advanced analytical techniques. Maybe more importantly, advanced theoretical understanding is needed to still come to valid and valuable conclusions from real-world movement data.

This Research Topic was aimed at highlighting current research that improves our understanding of human movement and motor control in natural environments, i.e., where individuals, patients, athletes, or other groups of interest perform, explore, and interact under real-world conditions. This article Research Topic features 17 articles—thirteen original research articles, three reviews, and one theoretical article—spanning multiple Research Topic in human movement science, including motor control and motor competence assessment, motor learning and coaching, and new approaches for field-based assessments of movements in everyday life and sports. In over half of the original research articles, human movement was studied in out-of-the-laboratory settings with six studies including actual outdoor settings. The remaining five original research articles either inform future movement assessments in out-of-the-lab settings or expand our understanding of how motor development and changing environments affect the human movement system. The three review articles summarized combined evidence from laboratory-based and field-based investigations, while the theoretical article presented a system-theoretical model of human motor behavior. The remainder of this editorial will highlight specific findings of the included articles that best showcase how our current understanding of the human movement system may evolve or be challenged when studied out-of the lab in natural environments.

## Motor control and competence assessment in natural environments

The two studies by Ng and Button and van Duijn et al. focused on the importance of assessing motor skills under ecologically-valid conditions. The report by van Duijn et al. dealt with aquatic motor skills and argued for the necessity of including open water education in water safety competence assessments. Based on two pilot studies, the authors showed that pool-based assessments may fall short in replicating the motor and cognitive skills needed by learners to prevent accidents in open water. The authors suggested that future assessment batteries for water safety competence should include open water environments wherever possible while ensuring learner safety. Similarly, the report by Ng and Button challenge the validity of traditional assessments of general motor competence in children because these assessments are based on isolated movement tasks (e.g., running, jumping, throwing) that are performed out-of-context. Instead, the authors proposed to use active video gaming technology to assess children’s motor competence in virtual environments that better mirror the capacity of the movement system to perform in and interact with natural and changing environments. Ng and Button presented a new video game-based instrument for movement competence assessment and underscored its internal validity for measuring four underlying movement competence constructs, i.e., stability, object-control, locomotion, and dexterity.

With a specific focus on the movement stability construct, the original research article by Angulo-Barroso et al. investigated how landing movement strategies evolve with age in three to nine year-old children. The authors demonstrated that landing strategies were influenced by the age of the children but not the presence or absence of an unexpected additional task (e.g., run to the left) following landing. They concluded that at the age of 4-5 years old, children go through a critical development phase regarding landing strategies characterized by the integration of more precise feedforward control mechanisms to modulate landing joint stiffness. Further, they argued that targeted practice of landing at this age and within natural environments, such as playgrounds, may facilitate this development step and help reduce the risk of landing injury in the long-term.

The reports by Xie et al. and by Huang et al. investigated the interaction of the movement system with its environment during various locomotion activities. Xie et al. demonstrated the influence of surface compliance on ankle joint dynamics during walking; specifically, that the control system adjusts ankle joint stiffness according to the surface compliance to maintain gait stability. Huang et al. used principal component analysis to describe, quantify, and compare lower limb movement patterns between a range of everyday locomotion tasks in varying environments. Using a movement similarity score, the authors categorized movements into three clusters (C1: walking, running, sitting-down, C2: hopping, C3: turning). Movements within these clusters can be reconstructed through combining basic kinematic synergies shared across clusters and cluster-unique kinematic synergies indicative of a modular motor control strategy. The authors suggested that the presented analytical framework can facilitate the assessment of real-world locomotion skills with specific application in rehabilitation and treatment planning.

## Motor learning and coaching

Articles in this category expanded our understanding of 1) how motor learning outcomes are influenced by the learning task, learning environment, and learning conditions, and 2) how classical views in the motor learning domain may not hold outside of the laboratory.

At the most basic level, Moriyama et al. used a virtual ball-kicking task to confirm that motor adaptation to visual inputs follows similar processes in the lower limb compared to what is well known for the upper limb. At a more translational level, two review articles by Zhao et al. and by Teraz et al. focused on cognitive aspects of motor learning in athletic and elderly populations. Specifically, Teraz et al. used a systematic review to investigate whether exercise interventions with added cognitive tasks, i.e., “motor-cognitive training,” were more efficacious in improving mobility outcomes compared to exercising alone in elderly populations. They concluded that “motor-cognitive training”—particularly exergaming interventions—only lead to larger improvements in mobility than exercise alone when mobility assessments were based on multicomponent tasks such as the timed-up-and-go test rather than simple walking tasks. The authors assumed that this is due to higher ecological validity of multicomponent mobility tasks. In the athletic context, Zhao et al. systematically reviewed studies to assess the effect of video-based training on anticipation and decision-making in football. Based on ten included studies, the authors confirmed that football players demonstrate improved anticipation and decision-making during both standardized computer-based tests and during open play. Interestingly, the authors noted that video-based training for decision-making skills may be most effective if delivered in a “first-person perspective”, trying to imitate the real-world, natural environment as closely as possible for the learner, e.g., through virtual reality.

Collins et al. as well as Magelssen et al. studied motor learning and coaching at the implementation level in field-based scenarios. The study by Magelssen et al. is a good example for the disconnect that may arise when applying laboratory-derived motor learning theories to real-world learning. The authors compared the effects of an interleaved practice scheme (learners frequently switch between different learning tasks) and blocked practice scheme (learners sequentially go through each learning task) on skiing speed in slalom ski racers. While laboratory-derived theories would predict that interleaved practice leads to better performance during the skill retention phase, this prediction could not be confirmed for real-world learning of a complex skiing task. Among others, the authors suggest the absence of the expected effect to result from the high skill level of their participants and the continuous nature of the skiing task, which is in contrast to the discrete skills typically studied in laboratory experiments. Finally, Collins et al. asked the unusual but innovative question of how motor learning evolves in the absence of a prescribed learning strategy, i.e., in the absence of a coach. Specifically, they studied learning practices in skateboarders who are used to perform and train in coach-free environments. The authors demonstrated that skateboarders utilize a broad range of learning strategies that can be connected to elements from different and often contrasting theories on effective motor learning (e.g., cognitive vs. ecological). The authors suggest that, rather than advocating for the use of one specific coaching approach, effective coaching should be guided by the needs and preferences of the learners. Studying behavior of performers in coach-free real-world environments can help inform the development of such learner-centered coaching approaches.

## New approaches for field-based human movement assessments

Contributions in this section presented novel approaches to quantify and/or analyze human movement in natural environments either for everyday or sports activities. One of the most fundamental variables to quantify real-world human movement is walking speed. Reggi et al. tested the validity of a GNSS-based real time kinematic (RTK) receiver to measure walking speed in a real-world outdoor setting. The authors demonstrated that RTKs improve the validity of walking speed estimates over standard GNSS-based estimates given the ability of RTKs to cope with poor sky visibility. Going a step further, van Andel et al. and Sharma and Rombokas explored analytical approaches to investigate full-body motor control strategies during locomotion in natural environments based on inertial-sensor based motion capture. Sharma and Rombokas compared different approaches to assess the “complexity” of lower limb movements during various ambulatory conditions, such as walking forward and backward, sidestepping, and unconstrained navigating through indoor environments. The authors showed that a range of common approaches to asses “movement complexity,” i.e., dimensionality, step-to-step variability, and non-linear measures of system dynamics, lead to different conclusions regarding “movement complexity” for most conditions. Practical recommendations were derived, including the suggestion to avoid the term “complexity” and use the specific term for the measured construct. Focusing on the dimensionality aspect of “movement complexity,” van Andel et al. investigated the influence of the measurement system (optical vs. inertial motion capture) and analysis approach (group-based vs. individual) on the dimensionality and temporal structure of postural changes during walking. Specifically, they used a principal component analysis (PCA) to structure the full-body motion into “principal movements” (PMs) and determined the internal consistency of PM-related outcomes between measurement systems and analysis approaches. Based on a high internal consistency for all lower-order PMs, i.e., those PMs that explain >95% of the total movement variance, van Andel et al. concluded that full-body inertial motion capture is suitable for studying movement dimensionality and basic postural variations outside of the laboratory. Further, they provided recommendations for avoiding potential pitfalls when quantifying movement dimensionality from either group-based or individual-based analyses of walking data.

Following a similar data Research Topic and analysis approach, but with application in sports science, Debertin et al. presented a proof of concept for a new method to quantify technique in alpine skiing. Typically, the output of a PCA is data-driven, i.e., defined by the movement to be analyzed. Debertin et al. proposed a new analysis framework where the PCA output is tailored to yield principal component axes for the assessment of essential technique elements of downhill skiing. This was achieved by curating a PCA input data set that included extreme variations of those technique elements performed by expert skiing instructors. The authors confirmed that their analysis framework can successfully discriminate between downhill skiing techniques and skill levels underscoring the potential use of this approach in technique training in skiing and beyond. Staying within the realm of downhill skiing but motivated by injury prevention, Heinrich et al. presented a novel approach for estimating joint moments of the lower limb and lower back during turning maneuvers. Specifically, they presented a three-dimensional musculoskeletal skier model, which tracks experimental kinematic data of a turning maneuver in a forward dynamics optimization framework. Heinrich et al. demonstrated an accurate reconstruction of the experimental data and reported joint moments in physiologically feasible ranges. They highlighted several advantages of this optimization approach compared to the classical inverse dynamics approach for estimating joint moments, e.g., that the described framework only requires kinematic inputs but no information about external forces, which makes it a feasible approach for analyzing actual racing and training runs. Finally, Zandbergen et al. presented lower limb running mechanics over the course of a marathon using an inertial motion capture system. The authors described a regression-based approach to estimate impact-related variables (e.g., tibial acceleration) during running that have been corrected for the influence of running speed and stride frequency. Zandbergen et al. highlighted scenarios where the analysis of uncorrected impact-related variables could lead to invalid recommendations for runners trying to improve their technique.

## Theoretical considerations

Last but not least, Petryński et al. offered theoretical insight regarding internal mechanisms of human motor behavior. Grounded in Nikolai Bernstein’s fundamental work on motor control, the authors presented a system-theoretical model of motor behavior, including two sub-systems: an information processing sub-system (from sensory reception to motor execution) and a sub-system containing the associated functional and operational modalities. The presented model and the authors’ arguments partially supported the motivation of this Research Topic: When studying the human movement system, its behavior can only be understood in its entirety if all information processing steps involved in a motor action—as they occur in natural environments—are considered or at least appreciated by the observer.

## Summary and conclusion

This Research Topic combines original research findings from diverse experimental settings with theoretical considerations on the behavior of the human movement system in natural and real-world environments. The included articles highlight the versatility of the movement system when humans are navigating on varying surfaces and through varying environments. Its versatility may be the pinnacle feature of the human movement system and if motor competence assessments are to capture this central feature, they must be conducted under close to real-world conditions. Similarly, motor learning interventions are likely most effective when they target all perceptuo-motor integration modalities of the movement system needed to perform the task of interest, e.g., anticipation and decision-making in sports or multisensory perception in aging. Further, real-world motor learning should be guided by the preferences and needs of the individual learner and the real-world tasks and environments in which their learned activities take place. Finally, this Research Topic provided know-how on data Research Topic techniques (e.g., full-body inertial motion capture) and analysis protocols (e.g., principal component analysis) to assess the versatility and mechanics of the human movement system outside of the laboratory while avoiding potential pitfalls. In conjunction, this article Research Topic advances our understanding and skillset to study human movement and motor control in real-world environments and paves the way for more ecologically valid conclusions about how to improve human performance and quality of life.

